# Comparative Skin Transcriptome Analysis Identifies Candidate Genes Associated with Skin Responses in Hu Sheep Raised Under Different Regional Rearing Conditions

**DOI:** 10.3390/ani16101550

**Published:** 2026-05-19

**Authors:** Gaoyi Ouyang, Yifan Hu, Wenping Dong, Yaqin Wu, Peiling Wei, Xuefeng Lv, Weiting Xing, Wenxin Zheng

**Affiliations:** 1 College of Animal Science, Xinjiang Agricultural University, Urumqi 830052, China; 15730832642@163.com (G.O.); 15083313151@163.com (Y.H.); 2 Institute of Animal Husbandry Quality Standards, Xinjiang Academy of Animal Sciences, Urumqi 830000, China; 18394493921@163.com (W.D.); 18189636538@163.com (Y.W.); 15699193535@163.com (P.W.); lxf00700@163.com (X.L.); 13899853128@163.com (W.X.); 3 Xinjiang Uyghur Autonomous Region Academy of Animal Science, Urumqi 830011, China

**Keywords:** Hu sheep, skin tissue, regional rearing environment, low-temperature-related response, transcriptome, candidate genes

## Abstract

To identify candidate genes associated with skin tissue responses in Hu sheep raised under different regional rearing environments and to preliminarily explore their potential relevance to low-temperature-related environmental responses, this study used 1-year-old female Hu sheep raised in Anhui and Xinjiang as the experimental animals. Skin tissues were collected from the left scapular region, and their transcriptomic profiles were characterized by integrating histological analysis, RNA sequencing (RNA-seq), differential expression analysis, functional enrichment analysis, protein–protein interaction (PPI) network construction, and RT-qPCR validation. The results showed significant differences between the two groups in body weight, body length, body height, cannon circumference, rectal temperature, and ear temperature. Hematoxylin and eosin (H&E) staining indicated that the Xinjiang group exhibited a denser distribution of hair follicles, a relatively thicker dermis, and a more compact arrangement of collagen fibers, suggesting enhanced insulation-related skin characteristics. Transcriptome sequencing identified 295 differentially expressed genes (DEGs), including 193 upregulated and 102 downregulated genes. GO and KEGG enrichment analyses showed that these DEGs were mainly involved in immune and inflammatory responses, redox processes, extracellular matrix remodeling, and lipid and energy metabolism-related pathways, with significant enrichment in cytokine–cytokine receptor interaction, the chemokine signaling pathway, the NF-κB signaling pathway, glutathione metabolism, and drug metabolism–cytochrome P450. By further integrating PPI network analysis and functional annotation, *CXCL13*, *CCL2*, *FGF21*, *GPX3*, *CYP1A1*, *HSD11B1*, *CDO1*, and *STEAP4* were identified as candidate genes. RT-qPCR results showed that the expression trends of the selected genes were generally consistent with the RNA-seq results. Overall, this study revealed differences in phenotypic traits, skin histological structure, and transcriptomic characteristics between Hu sheep raised in different regions, providing preliminary molecular clues potentially associated with low-temperature-related environmental responses. Given the differences in geographic origin and rearing environments between the two groups, the findings should be interpreted as associative evidence of skin transcriptomic responses in Hu sheep under different environmental conditions—rather than as direct causal evidence that low temperature alone drove these transcriptomic differences.

## Simple Summary

Hu sheep are an important breed in China, but their skin responses to different regional environmental conditions remain poorly understood. Because the skin is the part of the body that first comes into contact with the outside environment, studying skin changes may help explain how sheep respond to low temperatures. In this study, we compared Hu sheep raised in a warmer area with those raised in a colder area. We found clear differences between the two groups in body traits, body temperature, and skin structure. Sheep from the colder area had denser hair follicles, thicker skin, and more compact skin tissue, which may help reduce heat loss. We also found differences in the activity of many genes involved in body defense, energy use, protection against cell damage, and skin structure. These findings suggest that skin responses of Hu sheep under different regional environments may involve both physical changes in the skin and molecular-level changes. This study provides useful information for improving the breeding, management, and welfare of sheep raised under diverse environmental conditions, including low-temperature regions.

## 1. Introduction

Hu sheep are an important indigenous sheep breed originating from the Taihu Lake region of China. They are characterized by high prolificacy, relatively rapid growth, early sexual maturity, and the typical wavy pattern of lamb pelts. Owing to these favorable traits, Hu sheep are of considerable value in mutton sheep production, germplasm resource conservation, and genetic improvement in China. In recent years, with advances in population genetics, whole-genome resequencing, and transcriptomic technologies, increasing research attention has been paid to the genetic structure, selection signatures, formation of economically important traits, and environmental adaptability of Hu sheep [1,2]. These studies have provided an important genetic and molecular basis for further elucidating the adaptive changes of Hu sheep under different ecological environments.

Cold environments are one of the important external factors affecting the production performance, health status, and welfare of ruminants. Under low-temperature conditions, animals generally need to maintain physiological homeostasis by increasing heat production, reducing heat loss, adjusting energy metabolism, and modifying behavioral activities. Previous studies have shown that low-temperature exposure can induce molecular responses related to shivering thermogenesis, non-shivering thermogenesis, lipid metabolism, redox homeostasis, and endocrine regulation, thereby further affecting animal growth and development, tissue structure, and environmental adaptability [3,4,5]. In recent years, increasing attention has been paid to the molecular response mechanisms of sheep under cold exposure. For example, transcriptome analysis has identified thermogenesis-related candidate genes in cold-exposed sheep [4], and changes in the expression of thermogenesis-related genes have been reported in the muscle tissue of cold-exposed lambs [5]. More recently, skin transcriptomic analysis has been used to investigate molecular responses in cold-exposed lambs [6], further suggesting that skin tissue plays an important role in low-temperature environmental responses.

The skin is an important organ that is directly exposed to the external environment and plays a key role in physical barrier function, protective defense, and thermoregulation. In sheep, skin structure and its appendages, including epidermal thickness, dermal composition, hair follicle density, hair follicle developmental status, collagen fiber arrangement, and sebaceous gland structure, may all influence wool formation, heat retention, and local tissue homeostasis. Previous studies have shown that hair follicle development, skin tissue structure, and extracellular matrix remodeling are closely associated with wool traits and environmental adaptability in sheep [7,8,9,10]. A higher hair follicle density and a thicker dermis may contribute to enhanced barrier function and insulation capacity of the skin and fleece system. Meanwhile, extracellular matrix remodeling, hair follicle development, and local metabolic regulation may also jointly participate in adaptive responses to changes in environmental temperature. Recent studies on sheep skin transcriptomes have further indicated that skin gene expression patterns are closely associated with thermotolerance, cold-exposure responses, hair follicle development, and changes in tissue structure [6,8,10,11].

RNA sequencing (RNA-seq), as a high-throughput technology for systematically characterizing gene expression profiles, has been widely applied in studies of livestock stress responses, metabolic regulation, skin development, and mechanisms of environmental adaptation. Systematic reviews have shown that transcriptomics has become an important approach for elucidating the complex mechanisms underlying environmental adaptation in livestock [12]. Meanwhile, comparative evaluations of RNA-seq differential expression analysis methods have indicated that, under certain sample size conditions, DESeq2 performs well in terms of false discovery rate control, statistical power, and result stability [13]. By integrating histomorphological observation, differential expression analysis, functional enrichment analysis, and candidate gene validation, animal responses to different environmental conditions can be more comprehensively characterized at the phenotypic, tissue-structural, and molecular levels. Although previous studies have investigated molecular responses in sheep under cold exposure, heat stress, or during hair follicle development [4,6,8,11], studies on skin tissue structure and transcriptomic responses of Hu sheep raised under different regional and climatic conditions remain relatively limited. In particular, integrated analyses linking body size and temperature-related traits, hair follicle distribution, dermal structure, and the skin transcriptome remain to be further expanded.

Therefore, in the present study, Hu sheep raised under different environmental conditions in Anhui and Xinjiang were used as the experimental animals. Body size and temperature-related measurements, skin histological observation, and RNA-seq analysis were integrated to compare differences between the two groups at the phenotypic, skin-structural, and transcriptomic levels. This study focused on histological characteristics related to skin barrier function and insulation capacity, including hair follicle density, dermal thickness, and collagen fiber arrangement. Further, it screened candidate genes and pathways potentially associated with low-temperature-related environmental responses, skin structural remodeling, local immune regulation, and energy metabolism. Considering the differences between the two groups in rearing region, climatic conditions, and long-term environmental exposure background, this study aimed to provide preliminary molecular clues for investigating skin-mediated environmental responses and low-temperature-related response processes in Hu sheep, rather than attributing the observed differences solely to low temperature.

## 2. Materials and Methods

### 2.1. Experimental Animals and Sample Collection

In this study, 30 healthy female Hu sheep were selected for each of Group A and Group B, and all samples were collected in March 2025. Group A consisted of local 1-year-old female Hu sheep raised at Anxin Husbandry in Guoyang, Anhui Province. Meanwhile, Group B consisted of 1-year-old female Hu sheep, originally introduced from Anhui and subsequently raised for a long time at Anxin Husbandry in Yili, Xinjiang. All body temperature measurements and skin sample collections were performed during a fixed period before morning feeding (08:00–10:00) to minimize the effects of circadian rhythm and feeding status on the results. At the time of sampling, the ambient temperature was approximately 13 °C in Anhui and −8 °C in Yili, Xinjiang. All sheep were fed the same formulated pelleted diet and had free access to feed and water.

Body size traits, body weight, rectal temperature, and ear temperature were recorded for all sheep. Subsequently, six female sheep were randomly selected from each group for skin sample collection. Before sampling, the hair at the left scapular region was clipped and shaved, and local anesthesia was administered using 2% lidocaine. Skin tissue samples were then collected using a skin biopsy instrument. After sampling, the wound was sutured, and antibiotic treatment was administered. The collected skin tissues were separately preserved in 4% paraformaldehyde and liquid nitrogen; fixed samples were used for histological observation, whereas samples preserved in liquid nitrogen were stored at −80 °C to prevent RNA degradation. All sampling procedures were conducted in strict accordance with the requirements of the Experimental Animal Welfare Ethics Committee of the Institute of Animal Husbandry Quality Standards, Xinjiang Academy of Animal Sciences, with the ethical approval number 20250324016.

### 2.2. Measurement and Statistical Analysis of Body Size and Temperature-Related Traits

Body weight, body length, body height, chest circumference, cannon circumference, rectal temperature, and ear temperature were measured in 30 sheep from each of Group A and Group B using a weighing scale, measuring tape, sheep measuring stick, rectal thermometer, and ear thermometer, respectively. All measurements were performed during a fixed period before morning feeding. Data are presented as the mean ± standard deviation (mean ± SD). Before statistical analysis, the Shapiro–Wilk test was used to assess the normality of each trait, and Levene’s test was used to evaluate the homogeneity of variances between groups. For normally distributed traits, between-group comparisons were performed using an independent-samples *t*-test; when variances were unequal, Welch’s corrected independent-samples *t*-test was applied. For multiple phenotypic traits, *p*-values were adjusted using the Benjamini–Hochberg method to control the false discovery rate (FDR). Differences were considered statistically significant at *p* < 0.05 or FDR < 0.05.

### 2.3. Preparation of Skin Sections

#### 2.3.1. Paraffin Sectioning

After fixation, skin tissue samples were trimmed and sequentially dehydrated in graded ethanol solutions, followed by clearing with xylene and paraffin embedding [8,9]. After complete solidification of the paraffin, the embedded tissues were sectioned into continuous slices of approximately 4 μm thickness using a paraffin microtome for subsequent analysis.

#### 2.3.2. Hematoxylin and Eosin (H&E) Staining

Paraffin sections were deparaffinized in xylene and rehydrated to water, stained with hematoxylin for 3–5 min, differentiated, and then blued under running tap water for approximately 10 min, followed by eosin staining for 2 min. The sections were subsequently dehydrated through graded ethanol, cleared in xylene, mounted with neutral balsam, and observed and photographed under a light microscope [8,9]. To ensure the representativeness of histological observation, at least three non-overlapping low-magnification fields and three non-overlapping high-magnification fields were selected from each skin sample for observation and image acquisition. Morphological features, including hair follicle distribution, dermal structure, collagen fiber arrangement, and skin appendages, were compared. The images shown in the figure are representative histological images of each group.

### 2.4. Transcriptome Library Preparation, Sequencing, and Preprocessing

Total RNA was extracted using TRIzol reagent (Thermo Fisher Scientific, Waltham, MA, USA) according to the manufacturer’s instructions. RNA concentration and purity were measured using a NanoDrop 2000 spectrophotometer (Thermo Fisher Scientific, Waltham, MA, USA), and RNA integrity was assessed using an Agilent 2100 Bioanalyzer (Agilent Technologies, Santa Clara, CA, USA). Libraries were constructed using the VAHTS Universal V5 RNA-seq Library Prep Kit (Vazyme, Nanjing, China) following the manufacturer’s protocol. Library sequencing was carried out by Huazhi Biotechnology Co., Ltd. (Changsha, China) on the Illumina NovaSeq 6000 platform (Illumina, San Diego, CA, USA) with paired-end reads of 2 × 150 bp. Raw sequencing data were processed using fastp to remove adapter sequences and low-quality reads [14], and the clean reads were then aligned to the sheep reference genome (GCF_016772045.1) using HISAT2 (v2.2.1) [15]. Gene-level raw counts were generated using HTSeq-Count (v0.11.2) [16] and used for differential expression analysis with DESeq2 (1.20.0) [17]. In contrast, TMM-normalized expression values were used only for visualization of expression patterns, including principal component analysis (PCA) and heatmap construction [18].

### 2.5. Identification of Differentially Expressed Genes and Functional Enrichment Analysis

To evaluate biological consistency among samples, principal component analysis (PCA) and hierarchical clustering were performed in R (v3.4.3) based on gene-level raw count data. Differentially expressed genes (DEGs) were identified using DESeq2 with the thresholds of false discovery rate (FDR) < 0.05 and |log2FoldChange| > 1 [17], and the results were visualized in R (v3.4.3). Functional annotation of the identified DEGs was performed using the DAVID database (Version 6.8) for Gene Ontology (GO) enrichment and Kyoto Encyclopedia of Genes and Genomes (KEGG) pathway analysis [19].

### 2.6. Construction of the Protein–Protein Interaction Network

Based on the identified differentially expressed genes, protein–protein interaction (PPI) analysis was performed using the STRING database (Version 11.5, http://string-db.org) [20]. Only DEGs recognized by the STRING database and annotated with protein interaction information were retained for subsequent network construction. The resulting PPI network was visualized using Cytoscape software (Version 3.10.3) [21], and node ranking was performed with the CytoHubba plugin based on the Degree algorithm [22]. A comprehensive evaluation framework was used to identify candidate genes for subsequent focused analysis. Candidate genes were required to meet the differential expression threshold of FDR < 0.05 and |log2FoldChange| ≥ 1; to be recognized by the STRING database and located within the main PPI network or major functional subnetworks; and to have functions associated with the major biological processes or pathways identified by GO/KEGG enrichment analysis, including immune and inflammatory regulation, energy metabolism, redox homeostasis, lipid metabolism, endocrine regulation, or tissue remodeling. In addition, evidence from the existing literature was considered to evaluate the functional representativeness of each candidate gene. The topological features of the PPI network and Degree values were used mainly for preliminary candidate gene screening and prioritization, rather than as direct evidence for functional validation.

### 2.7. Candidate Gene Selection and qRT-PCR Assay

Based on differential expression significance, PPI network analysis results, functional enrichment results, functional representativeness, and primer design feasibility, *FGF21*, *CDO1*, *CXCL13*, *GPX3*, and *STEAP4* were selected from the candidate differentially expressed genes for RT-qPCR validation to assess the consistency of expression trends with the RNA-seq results. Complementary DNA (cDNA) was synthesized using the SuperScript II Reverse Transcription Kit (Invitrogen, Waltham, MA, USA), with each 20 μL reaction containing 1 μg of total RNA. qRT-PCR was performed using ChamQ SYBR qPCR Master Mix (Vazyme, Nanjing, China) on a QuantStudio 6 Flex Real-Time PCR System (Applied Biosystems, Carlsbad, CA, USA). The amplification conditions were as follows: initial denaturation at 95 °C for 3 min, followed by 40 cycles of 95 °C for 10 s and 60 °C for 30 s. Three technical replicates were included for each sample. β-actin was used as the internal reference gene, and relative gene expression levels were calculated using the 2^−ΔΔCt^ method [23]. Data reporting and quality control were performed in accordance with the MIQE guidelines [24]. Primer sequences are listed in Table 1.

## 3. Results

### 3.1. Body Size and Temperature-Related Traits

To further evaluate phenotypic differences in Hu sheep raised under different regional rearing conditions, body size, body weight, and body temperature-related traits were first compared between the two groups. In March 2025, body size and temperature-related measurements were performed on 30 Hu sheep from each of Groups A and B. After normality and homogeneity-of-variance tests, between-group comparisons were conducted for body size, body weight, and body temperature-related traits. The results showed significant differences between Group A and Group B in body weight, body length, body height, cannon circumference, rectal temperature, and ear temperature, but no significant difference in chest circumference (Table 2). After correction for multiple comparisons, all traits except chest circumference remained statistically significant. Compared with Group B, Group A had lower body weight but greater body length, body height, cannon circumference, rectal temperature, and ear temperature.

### 3.2. Skin Histology

**Figure 1:**
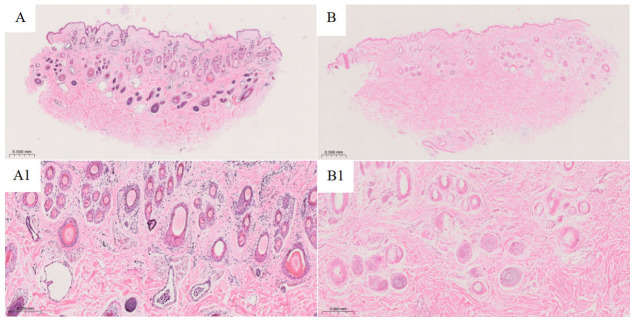
H&E staining of skin tissue in Group A and Group B Hu sheep. (**A**,**B**) Full-thickness skin structure of Group A and Group B under low magnification, respectively. (**A1**,**B1**) Corresponding dermal morphology of each group under high magnification.

Multiple non-overlapping fields were selected from each sample for histological observation and image acquisition. The images shown in Figure 1 represent the characteristic skin tissue morphology of each group. H&E staining revealed clear differences in hair follicle structure and dermal morphology between the two groups. Under low-magnification observation (Figure 1A,B), the skin of both groups consisted of the epidermis, dermis, and subcutaneous connective tissue. However, Group B showed a denser distribution of hair follicles, with multiple follicles arranged in clusters, and the number of hair follicles was markedly greater than that in Group A. In addition, the dermis of Group B was relatively thicker, with more compact collagen fiber arrangement and a denser overall tissue structure. In contrast, the dermal structure of Group A appeared relatively loose, with more sparsely distributed hair follicles. Under high-magnification observation (Figure 1A1,B1), Group B exhibited larger hair follicles, clearly defined hair bulbs, distinct follicular sheath layers, and relatively obvious sebaceous gland structures. In contrast, Group A showed fewer hair follicles, a more scattered distribution pattern, smaller follicular structures in some areas, and less distinct hair bulb morphology than Group B.

### 3.3. Quality Control of Sequencing Data

A total of approximately 710 million raw paired-end reads were generated by RNA-seq, with an average of approximately 59.49 million raw reads per sample, corresponding to a total of approximately 101.2 Gb of sequencing data. After quality control using fastp, an average of approximately 56.56 million high-quality clean reads was retained per sample, with the mean proportion of valid reads exceeding 94.56% and a mean GC content of 48.92%, indicating that the overall sequencing data were of high quality (Table 3).

### 3.4. Sample Correlation and Expression Pattern Analysis

**Figure 2:**
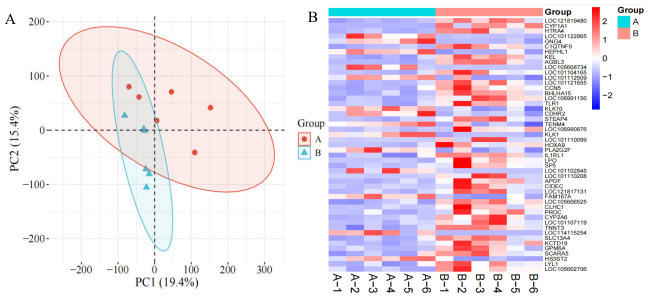
Principal component analysis (**A**) and hierarchical clustering heatmap of the top 30 differentially expressed genes (**B**) in skin tissue samples from Group A and Group B Hu sheep. Panel (**A**) shows the results of principal component analysis of the samples. In panel (**B**), each column represents one sample, and each row represents one gene; colors ranging from blue to red indicate low to high gene expression levels. A indicates samples from Group A, and B indicates samples from Group B.

Biological consistency and repeatability among samples were evaluated based on gene-level raw count data derived from clean reads. Principal component analysis (PCA) showed a clear separation between Group A and Group B in the principal component space, while samples within each group clustered closely, indicating strong within-group consistency (Figure 2A). To further characterize the expression patterns of differentially expressed genes, hierarchical clustering analysis was performed and visualized as a heatmap (Figure 2B). The results revealed clear differences in gene expression profiles between the two groups, and samples within each group clustered together, reflecting the good stability of the biological replicates. Some genes, such as *CYP1A1*, *HTRA4*, and *TLR1*, showed higher expression levels in Group B than in Group A, whereas other genes, including *KLK1*, *PLA2G2F*, and *IL1RL1*, were upregulated in Group A but expressed at lower levels in Group B. These findings indicate marked transcriptomic differences in the skin tissue of Hu sheep under different environmental conditions.

### 3.5. Identification of Differentially Expressed Genes

**Figure 3:**
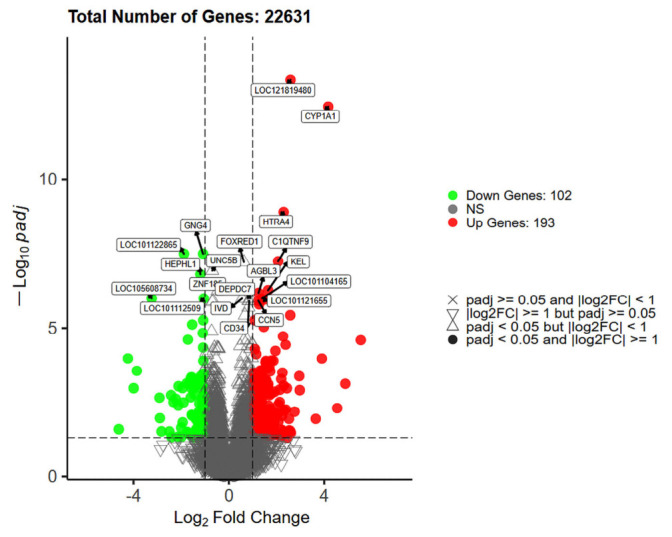
Volcano plot of differentially expressed genes in skin tissue between Group A and Group B Hu sheep. The x-axis represents log2FoldChange, and the y-axis represents −log10(FDR). Red dots indicate upregulated genes, blue dots indicate downregulated genes, and gray dots indicate genes without significant differential expression.

The Benjamini–Hochberg method was used to adjust *p*-values for multiple testing in order to control the false discovery rate (FDR). Differentially expressed genes (DEGs) were identified using the thresholds of FDR < 0.05 and |log2FoldChange| ≥ 1. In total, 295 DEGs were identified, including 193 upregulated genes and 102 downregulated genes, which were subsequently used for functional annotation and pathway enrichment analyses (Figure 3).

### 3.6. GO and KEGG Enrichment Analysis of Differentially Expressed Genes

**Figure 4:**
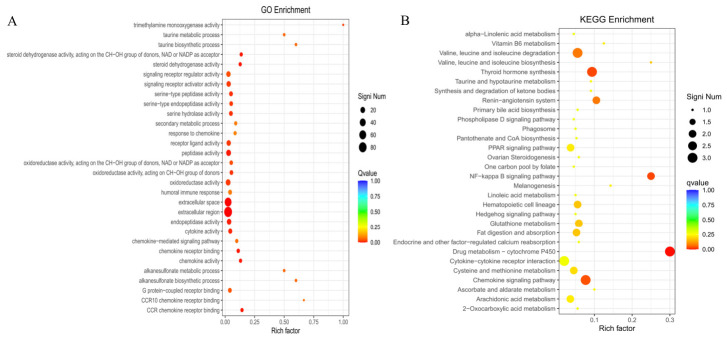
GO functional enrichment analysis (**A**) and KEGG pathway enrichment analysis (**B**) of differentially expressed genes in skin tissue from Group A and Group B Hu sheep.

As shown in Figure 4A, Gene Ontology (GO) annotation analysis indicated that the differentially expressed genes were involved in a wide range of biological functions, mainly including immune and inflammatory responses, extracellular structural organization, metabolic and redox processes, as well as protein degradation and tissue remodeling. In terms of immune- and inflammation-related functions, the DEGs were significantly enriched in chemokine activity, cytokine activity, chemokine receptor binding, chemokine-mediated signaling pathway, and humoral immune response. Regarding the extracellular environment and structural regulation, the enriched terms were mainly concentrated in the extracellular region and extracellular space. For metabolism- and redox-related processes, the DEGs were associated with oxidoreductase activity, NAD/NADP-dependent oxidoreductase activity, steroid dehydrogenase activity, taurine metabolism, and secondary metabolic processes. In addition, for protein degradation and tissue remodeling, peptidase activity, endopeptidase activity, serine-type peptidase activity, and serine hydrolase activity were all significantly enriched.

KEGG pathway analysis (Figure 4B) showed that the differentially expressed genes were significantly enriched in cytokine–cytokine receptor interaction, the chemokine signaling pathway, the NF-κB signaling pathway, glutathione metabolism, and drug metabolism–cytochrome P450. In addition, pathways related to branched-chain amino acid metabolism, arachidonic acid metabolism, and fatty acid metabolism also showed marked enrichment.

### 3.7. Protein–Protein Interaction Network Analysis

**Figure 5:**
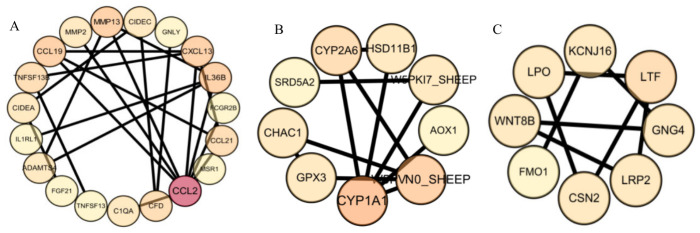
PPI subnetwork analysis of differentially expressed genes in skin tissue from Group A and Group B Hu sheep Panel (**A**) represents subnetwork 1 (immune and inflammatory regulation module); panel (**B**) represents subnetwork 2 (metabolism and oxidative stress regulation module); and panel (**C**) represents subnetwork 3 (hair follicle development and tissue morphogenesis module). The color intensity of each node represents the relative expression level of the corresponding gene, with darker colors indicating higher expression levels and lighter colors indicating lower expression levels. The connecting lines represent predicted protein–protein interactions among nodes.

Among the 295 identified differentially expressed genes, 67 genes were recognized by the STRING database and annotated with protein interaction information; therefore, they were included in the PPI network analysis. A protein–protein interaction network was constructed based on these 67 DEGs and visualized using Cytoscape. After removing isolated nodes, the final interaction network retained 36 interconnected genes and was divided into three main subnetworks: subnetwork 1 contained 19 nodes and 34 interaction edges (Figure 5A), subnetwork 2 contained 9 nodes and 12 interaction edges (Figure 5B), and subnetwork 3 contained 8 nodes and 10 interaction edges (Figure 5C). Nodes were further scored and ranked using the Degree algorithm in the CytoHubba plugin. Candidate genes for subsequent focused discussion were selected by integrating differential expression results, functional enrichment patterns, and functional representativeness. Because this network included only a subset of DEGs with available interaction annotations, the PPI analysis was used mainly for preliminary candidate gene screening and prioritization.

### 3.8. Candidate Genes and Their Differential Expression

**Figure 6:**
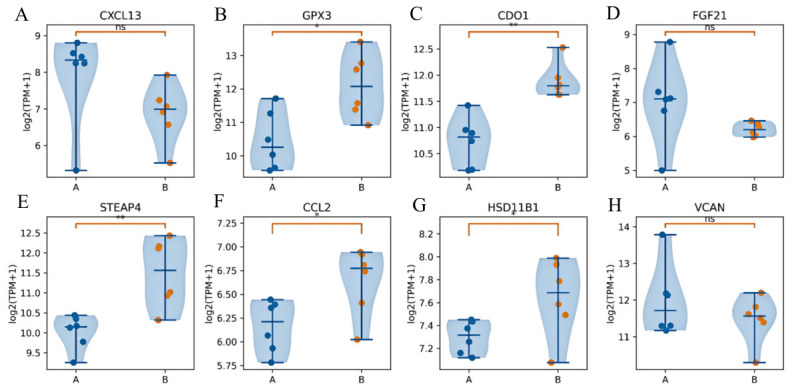
Expression distribution of candidate genes in skin tissue from Group A and Group B Hu sheep. (**A**) *CXCL13*; (**B**) *GPX3*; (**C**) *CDO1*; (**D**) *FGF21*; (**E**) *STEAP4*; (**F**) *CCL2*; (**G**) *HSD11B1*; (**H**) *VCAN*. Gene expression levels were visualized after log2 transformation. The violin plots show the distribution of gene expression, with overlaid scatter points representing expression values of individual samples and boxplots indicating the median and interquartile range. Between-group differences were analyzed using Student’s *t*-test, where * indicates *p* < 0.05, ** indicates *p* < 0.01, and ns indicates no significant difference.

Based on the differential expression results, PPI network topological features, GO/KEGG functional enrichment patterns, and evidence from the existing literature, eight candidate genes, including *CXCL13*, *CCL2*, *FGF21*, *GPX3*, *CYP1A1*, *HSD11B1*, *CDO1,* and *STEAP4*, were selected from the differentially expressed genes for further expression pattern analysis. These genes all met the differential expression screening criteria and were associated with major functional processes, including immune and inflammatory regulation, energy metabolism, redox homeostasis, endocrine regulation, or tissue remodeling. Violin plots were then generated to visualize the expression distribution of these candidate genes between Group A and Group B (Figure 6A–H). The results showed that all candidate genes were significantly differentially expressed between the two groups (*p* < 0.05).

### 3.9. qRT-PCR Validation

**Figure 7:**
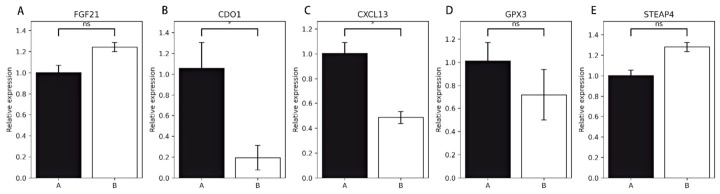
qRT-PCR validation of candidate genes. (**A**) *FGF21;* (**B**) *CDO1*; (**C**) *CXCL13*; (**D**) *GPX3*; (**E**) *STEAP4*. Relative expression levels were calculated using the 2^−ΔΔCt^ method, with Group A used as the control. Data are presented as mean ± standard error of the mean (mean ± SEM). Between-group differences were analyzed based on ΔCt values using Student’s *t*-test, where * indicates *p* < 0.05 and ns indicates no significant difference.

To further evaluate the reliability of the RNA-seq results, five differentially expressed genes, namely *FGF21*, *CDO1*, *CXCL13*, *GPX3*, and *STEAP4*, were selected for RT-qPCR validation (Figure 7A–E). These genes covered major functional processes, including immune regulation, energy metabolism, redox homeostasis, and amino acid/lipid metabolism. The RT-qPCR results showed that the expression trends of these five genes in the skin tissues of the two Hu sheep groups were generally consistent with the RNA-seq results, supporting the reliability of the sequencing data in terms of expression trends. However, the number of genes validated by RT-qPCR in this study was limited, and the validation was mainly used to assess the directional consistency between RNA-seq and RT-qPCR expression changes; therefore, it should not be regarded as evidence for the biological functions of the candidate genes.

## 4. Discussion

In this study, body size and temperature-related measurements, skin histological observation, and transcriptome sequencing were integrated to compare the skin tissue response characteristics of Hu sheep raised under different regional rearing conditions in Anhui and Xinjiang. The results showed clear differences between the two groups in several body size and temperature-related traits, as well as skin histological structure. The differentially expressed genes were mainly enriched in processes related to immune and inflammatory regulation, redox metabolism, lipid and energy metabolism, and extracellular matrix remodeling [25]. These findings suggest that skin tissues of Hu sheep raised under different regional rearing conditions may undergo transcriptomic changes associated with barrier function maintenance, local immune homeostasis, tissue structural remodeling, and metabolic regulation. Similarly, previous studies have shown that low-temperature, high-altitude, or other complex environmental conditions can induce transcriptomic and metabolic adjustments in multiple animal tissues, accompanied by coordinated changes in antioxidant capacity, energy substrate utilization, and immune status [26].

Body size, body weight, and body temperature-related traits are important phenotypic parameters for evaluating animal growth and development, production performance, and environmental adaptability. For ruminants exposed to low-temperature or complex environmental conditions, changes in body weight and body size may reflect differences in growth status, nutrient utilization, and energy allocation, whereas rectal temperature and ear temperature can, to some extent, reflect the ability to maintain core body temperature and the status of peripheral heat dissipation. Previous studies have shown that environmental stress can further affect growth performance and environmental adaptability in small ruminants by influencing feed intake, energy allocation, thermoregulation, behavioral responses, and metabolic status [3,4,5,27]. Recent research on heat-stress responses in Hu sheep has also shown that physiological indicators such as rectal temperature can be used to reflect thermoregulatory capacity under different thermal environments [28]. Therefore, the comparison of body size, body weight, and body temperature-related traits in this study provides a phenotypic background for interpreting the skin histological and transcriptomic differences in Hu sheep raised under different regional rearing conditions.

The histological results showed that the Xinjiang group had a denser distribution of hair follicles, relatively more mature follicular structures, increased dermal thickness, and more compact collagen fiber arrangement. These changes suggest that the skin tissue may improve insulation efficiency by enhancing fleece formation capacity and tissue compactness, thereby reducing heat loss under low-temperature conditions. Previous studies have shown that hair follicle developmental status, dermal structural integrity, and extracellular matrix remodeling are closely associated with animal responses to low-temperature environments [7,10]. In addition, studies on sheep hair follicle development and fleece traits have indicated that processes related to hair follicle formation, epidermal differentiation, and extracellular matrix regulation play important roles in maintaining skin structure and environmental adaptation [29,30]. The histological observations in this study were consistent with the enrichment of transcriptomic functions related to the extracellular region, proteolysis, and tissue remodeling, suggesting that skin structural remodeling may be an important component of the skin response of Hu sheep to different environmental conditions.

From the perspective of breed evolution and population genetic background, Hu sheep are generally considered to have originated from the Taihu Lake region of China and to have a certain genetic relationship with Mongolian sheep lineages. In recent years, whole-genome resequencing studies have shown that Hu sheep have a relatively distinct genetic basis in terms of genetic structure, selection signatures, and the formation of economically important traits, with related selection signals involving processes such as reproduction, growth, and immune response [2,31]. Broader sheep population genomic and landscape genomic studies have also indicated that sheep populations in different ecological environments may have developed differentiated genetic backgrounds and adaptive characteristics under long-term natural and artificial selection, and that genetic loci associated with bioclimatic variables such as temperature and precipitation may be involved in thermoregulation, immunity, and hair development [32,33,34,35]. Therefore, the environmental response characteristics observed in Hu sheep introduced and raised in different regions may be influenced not only by the current rearing environment but also by their breed evolutionary history and population genetic background. Since phylogenetic reconstruction or population structure analysis was not performed in this study, these considerations should be regarded only as background information for interpreting the results and not as direct evidence that the observed differences were caused by low temperature alone.

GO and KEGG enrichment analyses showed that immune- and inflammation-related functions were prominent in the comparison of skin tissues between the two Hu sheep groups, mainly involving chemokine activity, cytokine activity, chemokine-mediated signaling pathways, cytokine–cytokine receptor interaction, and the NF-κB signaling pathway. These results suggest that transcriptomic differences in skin tissue may be accompanied by changes in the local immune microenvironment. However, immune-related enrichment may also reflect general environmental stress, skin barrier status, or tissue remodeling rather than a low-temperature-specific response. Therefore, these immune-related changes are more appropriately interpreted as possible functional associations involved in local immune homeostasis under different environmental exposures. Recent studies on cold-stressed goats also suggest that immune regulation may interact with metabolic and microbial changes during environmental responses [36]. Therefore, these immune-related changes are more appropriately interpreted as local immune homeostatic regulation in Hu sheep skin under different environmental exposures, rather than as a specific mechanism driven by low temperature alone.

Among the candidate genes, *CXCL13* and *CCL2* were retained as immune-related candidates because they were differentially expressed, located in the PPI network, and functionally consistent with the immune-related enrichment results. Based on previous studies, the CCL2/CCR2 axis is associated with monocyte and macrophage recruitment, inflammatory regulation, fibrosis progression, and tissue repair [37], whereas CXCL13 is involved in immune cell positioning and inflammatory microenvironment formation [38]. In the present study, the differential expression of these genes may indicate changes in the local immune microenvironment of skin tissue. However, because immune cell infiltration, inflammatory factor protein levels, and spatial localization were not examined, their roles should be regarded as possible functional associations rather than confirmed mechanisms.

In addition to immune-related changes, metabolic and redox-related pathways may also be associated with skin responses under different environmental conditions. In this study, DEGs were enriched in glutathione metabolism, drug metabolism–cytochrome P450, arachidonic acid metabolism, and branched-chain amino acid metabolism, suggesting possible metabolic adjustment in skin tissue. Previous studies have shown that low-temperature exposure can induce transcriptomic and metabolic remodeling in metabolically active tissues, accompanied by changes in antioxidant capacity, energy substrate utilization, and immune status [25,26]. Among the related candidate genes, *FGF21* and *STEAP4* are associated with lipid mobilization, substrate utilization, mitochondrial function, and thermogenic regulation [39,40], whereas *GPX3* is related to antioxidant defense [41]. *CYP1A1*, *HSD11B1*, and *CDO1* may be associated with xenobiotic metabolism, endocrine stress regulation, and sulfur-containing amino acid metabolism, respectively [42,43,44]. These findings suggest possible functional links between the identified genes and energy metabolism, redox balance, and local tissue homeostasis. However, because protein expression, metabolite levels, and functional assays were not examined in this study, these interpretations should be considered literature-supported associations rather than experimentally verified mechanisms.

Taken together, the present study revealed functional associations among phenotypic traits, skin histological structure, and transcriptomic profiles. The denser hair follicle distribution, relatively thicker dermis, and more compact collagen fiber arrangement observed in the Xinjiang group were consistent with the enrichment of DEGs related to immune and inflammatory regulation, energy metabolism, redox homeostasis, and extracellular matrix remodeling. These findings suggest that the skin response of Hu sheep to different environmental conditions may involve coordinated changes in skin structure and molecular regulation, which is consistent with previous skin transcriptomic evidence showing that genes related to immunity, endocrine regulation, and hair follicle development participate in skin biology and environmental adaptation-related processes [45]. Using an integrated evaluation framework based on differential expression, PPI network position, functional enrichment, and literature evidence, *CXCL13*, *CCL2*, *FGF21*, *GPX3*, *CYP1A1*, *HSD11B1*, *CDO1*, and *STEAP4* were selected as candidate genes for further consideration. However, these results should be interpreted as associative evidence of possible functional links rather than direct causal or experimentally verified mechanisms.

Several limitations should be considered when interpreting these findings. First, this study was an observational comparison conducted under natural rearing conditions, and the differences between the two groups were not limited to ambient temperature. Although both groups were fed the same formulated diet, regional climate, housing microenvironment, management details, long-term environmental exposure, and potential genetic or epigenetic differences may also have influenced the observed transcriptomic patterns. Second, the RNA-seq sample size was relatively limited, with six biological replicates per group, which may reduce statistical power and affect the stability of DEG identification. In addition, RT-qPCR validation included only five genes and was mainly used to assess consistency of expression trends rather than functional validation. Finally, functional enrichment was mainly based on DAVID, and the PPI network included only DEGs with available interaction annotations; therefore, pathway interpretation and candidate gene prioritization should be considered preliminary. Finally, the functional enrichment analysis was mainly based on DAVID, and the PPI network included only DEGs with available interaction annotations; therefore, pathway interpretation and candidate gene prioritization should be considered preliminary. Because protein-level validation, metabolite measurements, immune cell localization, and functional assays were not performed, the proposed links among immune, metabolic, and structural pathways should be interpreted as possible functional associations. Future studies with larger sample sizes, independent populations, cross-validation using additional enrichment tools, and protein-level or functional experiments are needed to further validate these findings.

## 5. Conclusions

This study integrated skin transcriptome sequencing, body size and temperature-related measurements, and histomorphological observation to compare Hu sheep raised under different regional rearing conditions in Anhui and Xinjiang. Differences were observed between the two groups in several body size and temperature-related traits, hair follicle distribution, dermal structure, and skin gene expression profiles. A total of 295 DEGs were identified, and the enriched pathways were mainly associated with immune regulation, energy metabolism, redox homeostasis, and extracellular matrix remodeling. By integrating differential expression analysis, functional annotation, and PPI network analysis, *CXCL13*, *CCL2*, *FGF21*, *GPX3*, *CYP1A1*, *HSD11B1*, *CDO1*, and *STEAP4* were preliminarily identified as candidate genes. These findings provide preliminary molecular clues for understanding skin responses of Hu sheep to different regional rearing environments and low-temperature-related environmental conditions. Given the observational design and limited RNA-seq sample size, these candidate genes should be interpreted as associative findings requiring further validation in larger cohorts, independent populations, and controlled experimental models.

## Figures and Tables

**Table 1:** Primer sequences used for qRT-PCR in the present study.

Gene	Primer Sequence (5′-3′)	Primer Type	Product Size (bp)
*Actin-β-F*	CCACCGCAAATGCTTCTAGG	Forward Primer	206
*Actin-β-R*	AACCGACTGCTGTCACCTTC	Reverse Primer	
*FGF21-F2*	CTGCACTTTGACCCCAAAGC	Forward Primer	78
*FGF21-R2*	GGTCTCCGACTGGTAGACAT	Reverse Primer	
*CDO1-F2*	CCTGGCCTGACAAGAAATCC	Forward Primer	169
*CDO1-R2*	CATGTGTCAAAAGGCGGACT	Reverse Primer	
*CXCL13-F4*	GTCTGGATGAACAAAAAGCCAGTT	Forward Primer	131
*CXCL13-R4*	CAGGCACTCCTTTTCTTAACCAGT	Reverse Primer	
*GPX3-F1*	CCAGCTGTTTGAGAAAGGCG	Forward Primer	145
*GPX3-R1*	CCGGATGTCATGGACCTTCA	Reverse Primer	
*STEAP4-F1*	ATGCAGCCCAGAAATCTGACA	Forward Primer	155
*STEAP4-R1*	GTACTCTGCGTTTGACTCTGGA	Reverse Primer	

F indicates the forward primer, R indicates the reverse primer, and bp indicates the amplicon size.

**Table 2:** Comparison of body size and temperature-related traits between Group A and Group B Hu sheep.

Trait	Group B	Group A	*p*-Value
Body weight (kg)	52.50 ± 6.96	44.10 ± 8.34	8.50 × 10^−5^
Body length (cm)	57.00 ± 3.82	62.65 ± 4.99	8.00 × 10^−6^
Body height (cm)	70.62 ± 4.14	73.22 ± 4.38	0.0215
Chest circumference (cm)	88.49 ± 4.63	88.73 ± 4.19	0.8340
Cannon circumference (cm)	7.92 ± 0.61	9.00 ± 0.48	2.65 × 10^−10^
Rectal temperature (°C)	39.18 ± 0.38	39.59 ± 0.26	1.10 × 10^−5^
Ear temperature (°C)	35.17 ± 0.57	35.97 ± 1.34	0.0046

Data are presented as mean ± standard deviation (mean ± SD); *n* = 30 animals per group. Before analysis, normality and homogeneity of variance were assessed using the Shapiro–Wilk and Levene’s tests, respectively. Between-group comparisons were performed using an independent-samples *t*-test, with Welch’s correction applied when variances were unequal. *p*-values for multiple phenotypic traits were adjusted using the Benjamini–Hochberg method, and FDR < 0.05 was considered statistically significant.

**Table 3:** Quality statistics of skin transcriptome sequencing data from Group A and Group B Hu sheep.

Sample ID	Raw Reads	Clean Reads	Clean Q30 (%)	Clean GC (%)	Valid (%)
A-1	52,123,040	49,563,184	96.96	48.59	94.59
A-2	57,536,188	55,340,578	97.34	48.03	95.82
A-3	59,998,840	57,671,522	97.28	49.08	95.73
A-4	49,913,070	46,515,366	96.43	49.61	92.5
A-5	63,074,870	59,015,376	96.56	49.22	92.82
A-6	75,906,452	70,377,248	96.28	48.74	91.99
B-1	56,010,172	53,911,298	97.27	49.58	95.76
B-2	80,788,856	77,492,960	97.25	48.79	95.47
B-3	61,106,840	58,708,002	97.3	48.23	95.64
B-4	59,494,642	56,927,634	97.06	49.13	95.26
B-5	57,882,098	55,209,686	96.99	49.34	94.87
B-6	40,083,466	38,031,938	96.83	48.75	94.37

Raw reads indicate the original sequencing reads; clean reads indicate the high-quality reads retained after quality control; Q30 represents the proportion of bases with a quality score greater than 30; GC content indicates the proportion of guanine (G) and cytosine (C) bases in the sequencing data.

## Data Availability

The raw RNA-seq sequencing data generated during this study have been deposited in the NCBI Sequence Read Archive (SRA) under BioProject accession number PRJNA1455275. Additional data supporting the conclusions of this article are available from the corresponding author upon reasonable request.

## Abbreviations

The following abbreviations are used in this manuscript:

DEGs	Differentially Expressed Genes
PCA	Principal Component Analysis
GO	Gene Ontology
FDR	False Discovery Rate
KEGG	Kyoto Encyclopedia of Genes and Genomes
cDNA	Complementary DNA
qRT-PCR	Quantitative real-time polymerase chain reaction
